# Barbed suture versus traditional suture in primary total knee arthroplasty: A systematic review and meta-analysis of randomized controlled studies

**DOI:** 10.1097/MD.0000000000019945

**Published:** 2020-05-22

**Authors:** Pengbiao Li, Wenhui Zhang, Yanyan Wang, Jinlong Li, Peijing Yan, Shifang Guo, Jie Liu, Kehu Yang, Zongru He, Yaowen Qian

**Affiliations:** a Gansu Provincial Hospital, Lanzhou, Gansu; b School of Clinical Medical Sciences, Ningxia Medical University; c People's Hospital of Ningxia Hui Autonomous Region, Yinchuan; d Institute of Clinical Research and Evidence-Based Medicine, Gansu Provincial Hospital; e Evidence-Based Social Science Research Center, Lanzhou University; f Key Laboratory of Evidence Based Medicine and Knowledge Translation of Gansu Province; g Evidence-Based Medicine Center, School of Basic Medical Sciences, Lanzhou University, Lanzhou, China.

**Keywords:** barbed suture, randomized controlled trials, total knee arthroplasty, traditional suture

## Abstract

**Background:**

Barbed suture has been widely used in some surgical fields, and it has achieved good results, but the application in total knee arthroplasty is still controversial.

**Objective:**

Literature is collected for statistical analysis so as to provide evidence for the use of barbed suture in Total knee arthroplasty.

**Methods:**

We searched PubMed, the Cochrane library and EMBASE database for randomized controlled trials (RCTs) using barbed suture and conventional suture to close incisions after primary total knee arthroplasty, and the retrieval time was from July 2019 to the establishment of the database. Literature was screened according to inclusion and exclusion criteria, quality evaluation and data extraction were conducted for the final included literature, and statistical analysis was conducted using RevMan 5.3 software.

**Results:**

A total of six RCTs (826 knees) were included in our meta-analysis. The results showed that the re-negative conversion could shorten the wound closure time (MD –4.41, 95% CI −5.11 to −3.72, *P* < .00001) and reduce the wound closure total cost (MD –282.61, 95% CI –445.36 to –119.85, *P* = .0007) and acupuncture injury (RR 0.14, 95% CI 0.03–0.78, *P* = .02), and did not significantly increasing the incidence of complications (RR 0.80, 95% CI 0.05–0.96, *P* = .38) or suture breakages (RR 4.58, 95% CI 0.16−128.29, *P* = .37). There were no significant differences in ROM at postoperative 6 weeks and 3 months (MD −0.74, 95% CI −4.19 to 2.71, *P* = .67; MD −0.30, 95% CI −2.62 to 2.02, *P* = .80) and no significant differences in KSS at postoperative 6 weeks (MD –0.22, 95% CI –3.10 to 2.66, *P* = .88).

**Conclusions:**

Our study shows that barbed suture is a fast, low-cost, safe and effective suture method in total knee arthroplasty compared with traditional suture, we also need more literature and longer follow-up to confirm this conclusion.

## 1 Introduction

With the aging of the population gradually increasing, the incidence of knee disease is gradually increasing, so the number of people receiving total knee arthroplasty is increasing,^[1,2]^ total knee arthroplasty is one of the important methods for the treatment of end-stage knee disease, which can effectively correct and relieve pain and improve the quality of life of the patients.^[3,4]^ Joint closure technology is an important part of total knee arthroplasty, and good joint closure techniques prevent infection and increase patient satisfaction.^[5,6]^ The traditional method is to use interrupt technology, not only time-consuming, but also easy to cause infection.^[7]^ In recent years, a new suture technique known as barbed suture has been used in clinical,^[8]^ some reports indicate that the application of barbed suture in orthopedic and other surgeries has achieved good results,^[9,10]^ and there are many advantages, such as better waterproofing, higher cyclical tension,^[11,12]^ reduced surgical time, and cosmetic effects.^[8]^ However, there are other reports that the technique of barbed suture has the disadvantages of easy fracture and many complications.^[13,14]^ Therefore, the application of barbed suture in orthopedics, especially in total knee arthroplasty, is still controversial.

In addition, high quality meta-analysis has been increasingly regarded as one of the key tools for achieving evidence.^[15–17]^ The latest meta-analysis put total knee arthroplasty and total hip arthroplasty together for discussion.^[18]^ However, different surgical sites may lead to different clinical outcomes. In addition, there is new evidence in total knee arthroplasty. Therefore, we designed this study to obtain the final results through multiple steps including literature retrieval, literature screening, literature quality evaluation, data extraction, and statistical analysis, to provide evidence for the application of barbed suture in total knee arthroplasty.

## 2 Methods

### 2.1 Search strategy

The present study was conducted according to the Preferred Reporting Items for Systematic reviews and Meta-Analyses guidelines.^[16,19]^ A MeaSurement Tool to Assess systematic Reviews was used to assess methodological quality.^[20,21]^

Two researchers independently searched of the EMBASE, PubMed, and Cochrane Library databases up to July 2019 were performed. The search terms included “knotless” or “quill” or “fishbone” or “barbed” and “knee arthroplasty” or “knee replacement” or “joint arthroplasty” or “joint replacement” or “TKA” or “TKR,” the keyword and the free word are retrieved together, and the references of the identified studies were manually searched, the search was not limited by year. Then, we conducted literature screening, literature quality evaluation, data extraction, and statistical analysis.

### 2.2 Selection criteria

The inclusion criteria were as follows:

1. Randomized controlled trials (RCTs).
2. Patients in the study received primary total knee arthroplasty treatment and did not interfere with the clinical results of the remaining related diseases.
3. The study reported is a comparison of barbed suture and traditional suture.
4. At least one of the following: Key indicators
  (a) Wound closure time,
  (b) Wound closure total cost (contain material costs and the cost of operating room time),
  (c) Complications,
  (d) Knee Society Scores,
  (e) Knee range of motion, secondary indicators;
  (f) Suture breakages
  (g) Acupuncture injury.

The exclusion criteria were as follows:

1. duplicate articles;
2. case reports, reviews, meta-analysis, editorials, letters, Non-English, Non-Human, and cadaver experimental studies;
3. data that could not be extracted.
4. Reports that are not relevant to this study.

### 2.3 Date extraction

Two researchers independently extracted data from the included study: first author's name, date of publication, country, average age of the patient, sample size, patients’ gender, body mass index, follow-up time, incision length, key indicators, and secondary indicators. In the case of data loss, we tried to contact the corresponding authors for details, and if the two researchers disagree, we will seek the help of a third researcher.

### 2.4 Quality assessment

In order to assess methodological quality of each eligible study, two researchers independently assessed each included study using the Cochrane Collaboration tool for assessing the risk of bias.^[22]^ Including random sequence generation, allocation concealment, blinding of participants and personnel, blinding of outcome assessments, attrition bias, reporting bias, and other bias. Each question is answered with "yes," "no," and "unclear." The level of risk can be judged as "low risk," "high risk," "unclear risk." Any differences are resolved by discussion or by the corresponding authors.

### 2.5 Statistical analysis

Statistical analysis uses the RevMan 5.3 software provided by the Cochrane Collaboration Network. The heterogeneity between the studies uses *Q* test and *I*^2^ test,

1. If *P* > .1 or *I*^2^ ≤ 50%, we think there is no obvious heterogeneity between the included studies, we use the fixed-effect model to merge the data.
2. If *P* < .1 or *I*^2^ > 50%, we consider that there is heterogeneity among many results, and we use random effect model to combine data and analyze heterogeneous sources.

For continuous variables, we use mean difference (MD) with 95% confidence interval (95% CI). For classification variables, we use risk ratios (RR) with 95% CI. The test level was set to a 0.05, and the heterogeneity of clinical manifestations was analyzed by grouping analysis or sensitivity analysis, or only descriptive analysis.At the same time, we carried out sensitivity analysis; we removed the literature with poor quality from the included studies and conducted a meta-analysis again to compare whether there were significant differences between the combined effects before and after.

## 3 Results

### 3.1 Study selection

On systematic retrieval through electronic searches of the EMBASE, PubMed, and the Cochrane library, a total of 112 studies were obtained, a total of 34 articles were excluded because of duplicated, after reading the article title and summary, 62 articles were excluded because there was no obvious correlation or non-RCTs, after further reading the full-text, 10 articles were excluded

1. duplicated studies: n = 5,
2. studies date not extractable: n = 2,
3. non-human: n = 2,
4. non-English: n = 1.

**Figure 1:**
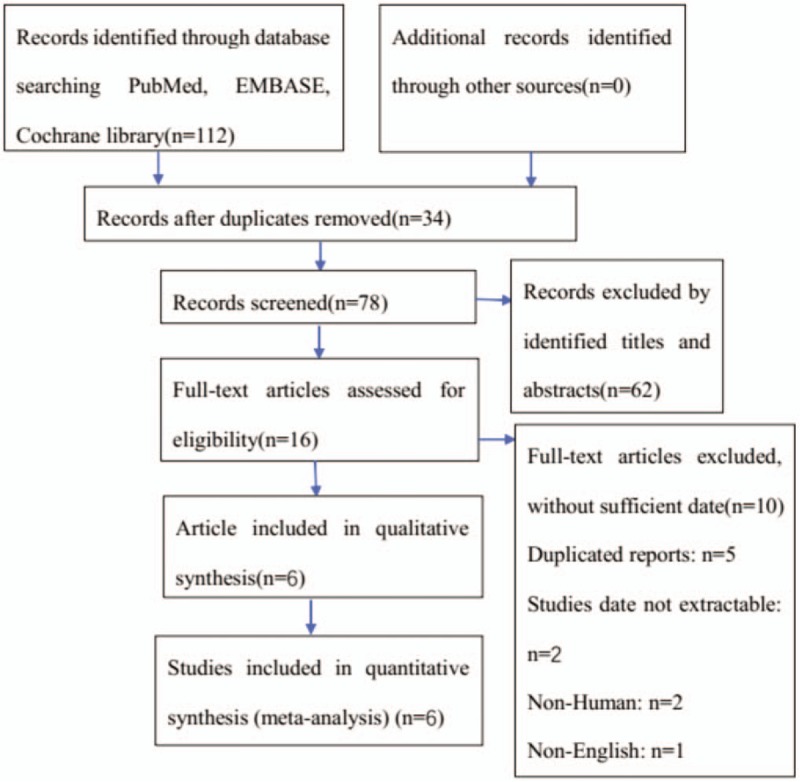
Flowchart of the literature search in the meta-analysis.

The last 6 articles meet the criteria and are included in the analysis (Fig. 1).

### 3.2 Characteristics of the included studies

Table 1 lists the features of the studies, which were published in six articles between 2012 and 2017, the last of which included a preliminary study on age. There was no difference in sex, body mass index, and other basic characteristics among groups. The relevant information and details of suture material are set out in Table 2.

**Table 1:**
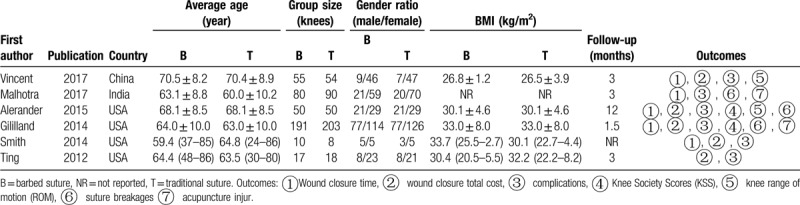
Study characteristics.

**Table 2:**
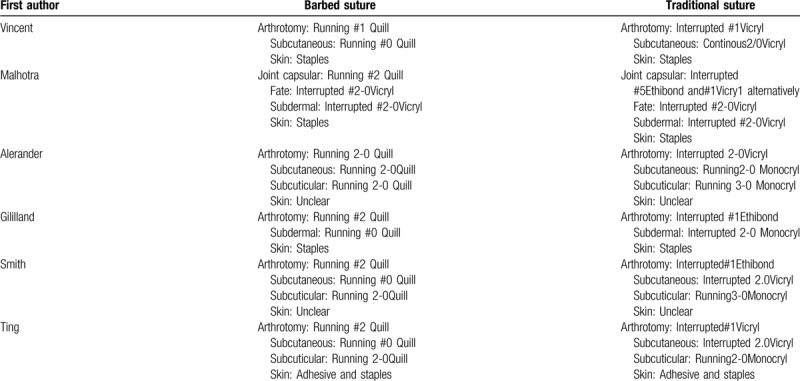
Details of the stitching materials and methods.

### 3.3 Risk of bias

**Figure 2:**
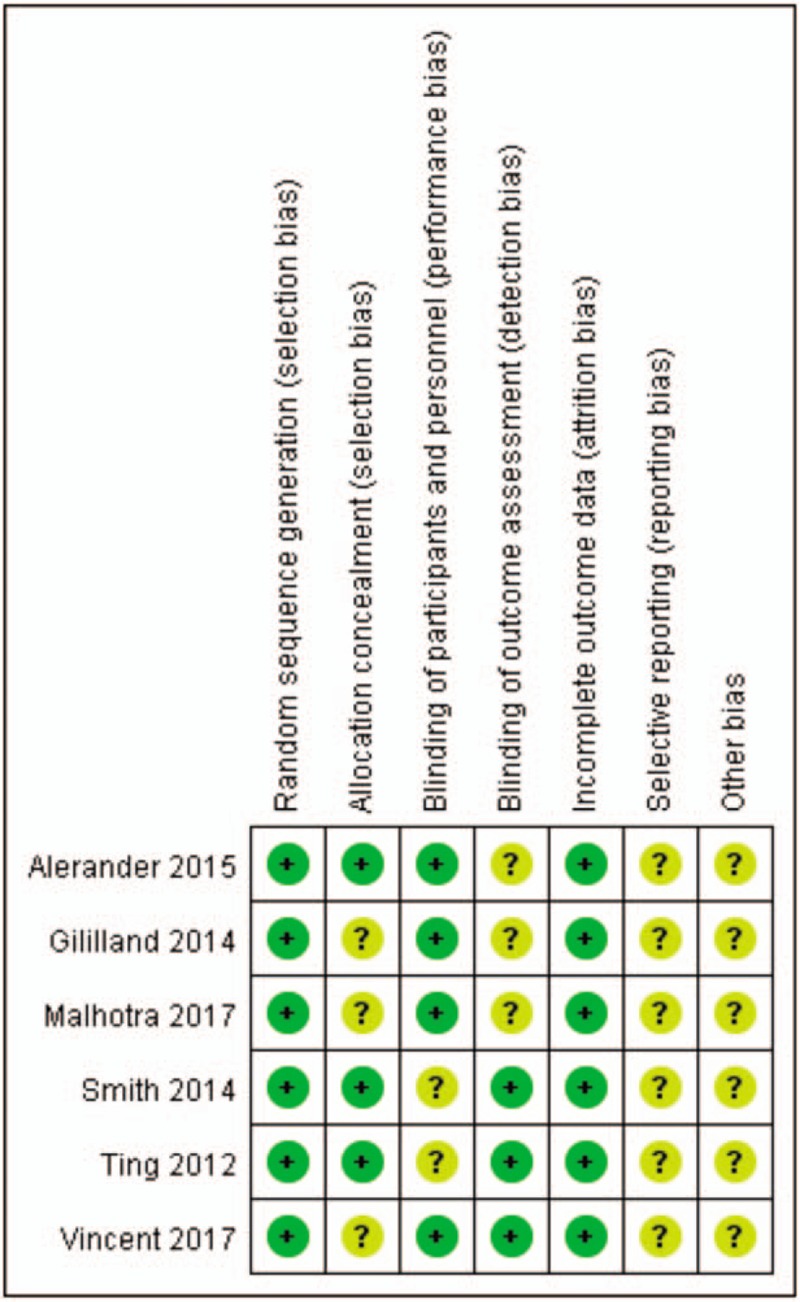
Summary of risk of bias for the included.

The results of the quality assessment are shown in Figure 2. Six studies adequately described the correct randomization, three studies showed adequate allocation, four studies described consistency in the assessment of results, and three studies described participants and personnel. All studies retain complete data on the results, in addition, reporting bias and other prejudices are not described in any included study.

### 3.4 Outcomes analysis

#### 3.4.1 Wound closure time

**Figure 3:**
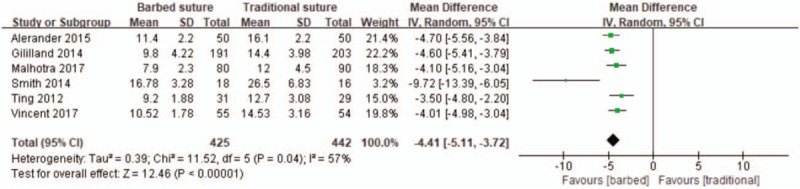
Forest plot on the assessment of total closure time.

All six studies reported the wound closure time^[23–28]^; which showed significantly shorter total closure time with barbed suture than traditional suture (MD –4.41, 95% CI −5.11 to −3.72, *P* < .00001; Fig. 3).

#### 3.4.2 Wound closure total cost

**Figure 4:**
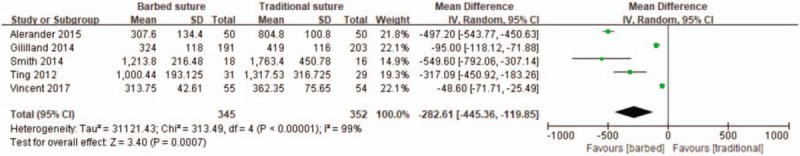
Forest plot on the assessment of wound closure total cost.

All six studies reported the total cost.^[23–28]^ However, one study did not say what the cost of operating room time.^[16]^ Full details regarding total cost are summarized in Table 3. In final, five studies were included which showed significantly shorter total closure cost with barbed suture than traditional suture (MD –282.61, 95% CI –445.36 to –119.85, *P < *= .0007; Fig. 4).

**Table 3:**
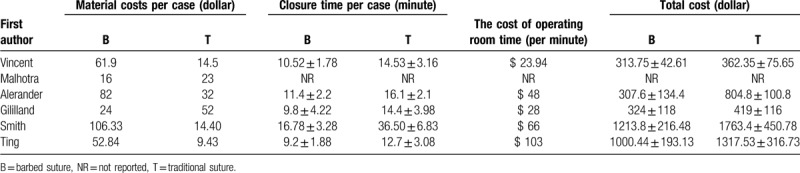
Details of the total cost.

#### 3.4.3 Complications

**Figure 5:**
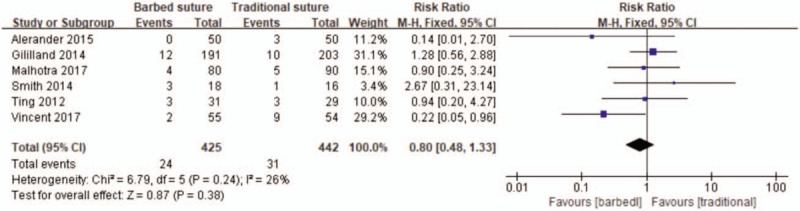
Forest plot on the assessment of complications.

All six studies reported the wound complications.^[23–28]^ Subsequently, no significant difference was detected in complications between the two groups (RR 0.80, 95% CI 0.05–0.96, *P* = .38; Fig. 5).

#### 3.4.4 Knee society scores

**Figure 6:**
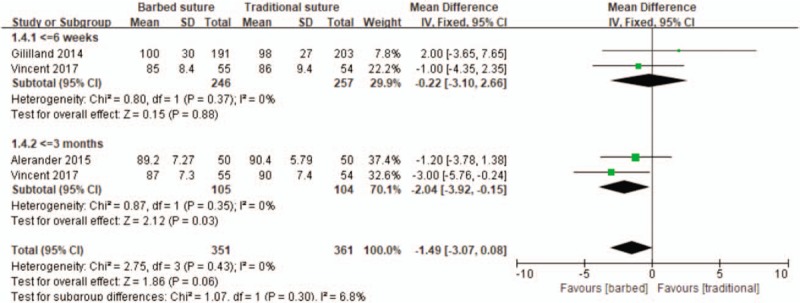
Forest plot on the assessment of Knee Society Scores.

There studies reported knee society scores at 6 weeks and 3 months after surgery.^[23,25,26]^ Therefore, we performed subgroup meta-analyses to compare the knee society scores based on the date. There were no significant heterogeneities among the subgroups (*P* = .37, *I*^2^ = 0%; *P* = .35, *I*^2^ = 0%; Fig. 6) and there were no significant differences between the two groups at postoperative 6 weeks (MD –0.22, 95% CI –3.10 to 2.66, *P* = .88). But at postoperative 3 months, the barbed suture group obtained a good knee society scores (MD –2.04, 95% CI –3.92 to –0.15, *P* = .03).

#### 3.4.5 Knee range of motion

**Figure 7:**
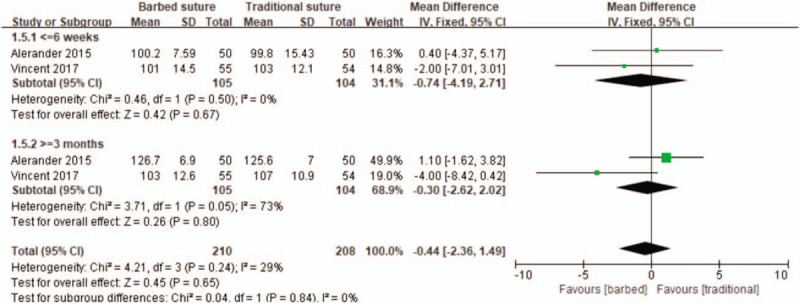
Forest plot on the assessment of Knee range of motion.

Two studies reported knee range of motion at 6 weeks and 3 months after surgery.^[23,25]^ Therefore, we also performed subgroup meta-analyses to compare the knee range of motion based on the date. There was no significant difference between the two groups at 6 weeks and 3 months after operation (MD −0.74, 95% CI −4.19 to 2.71, *P* = .67; MD −0.30, 95% CI −2.62 to 2.02, *P* = .80; Fig. 7).

#### 3.4.6 Suture breakages

**Figure 8:**
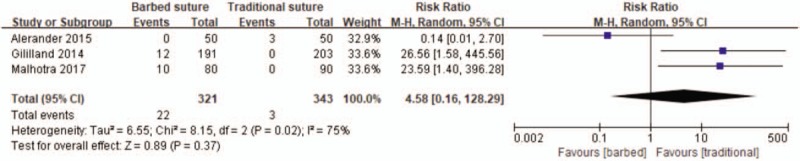
Forest plot on the assessment of suture breakages.

Three studies reported suture breakages in surgery,^[24–26]^ subsequently, three studies were included which showed more quantity with barbed suture, but this difference is not statistically significant (RR 4.58, 95% CI 0.16−128.29, *P* = .37; Fig. 8).

#### 3.4.7 Acupuncture injury

**Figure 9:**
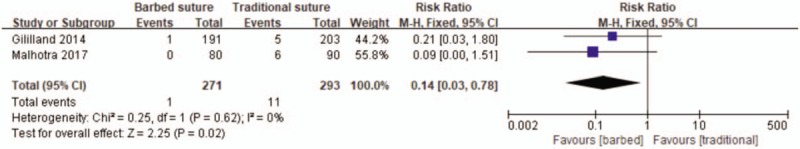
Forest plot on the assessment of acupuncture injury.

Two studies reported acupuncture injury in surgery.^[24,26]^ In final analysis, there were no significant heterogeneities among the two groups (*P* = .62, *I*^2^ = 0%; Fig. 9). The results of the two groups showed that the incidence of acupuncture injury was significantly lower than the traditional recurrence rate (RR 0.14, 95% CI 0.03–0.78, *P* = .02; Fig. 9).

#### 3.4.8 Publication bias and sensitivity test

**Figure 10:**
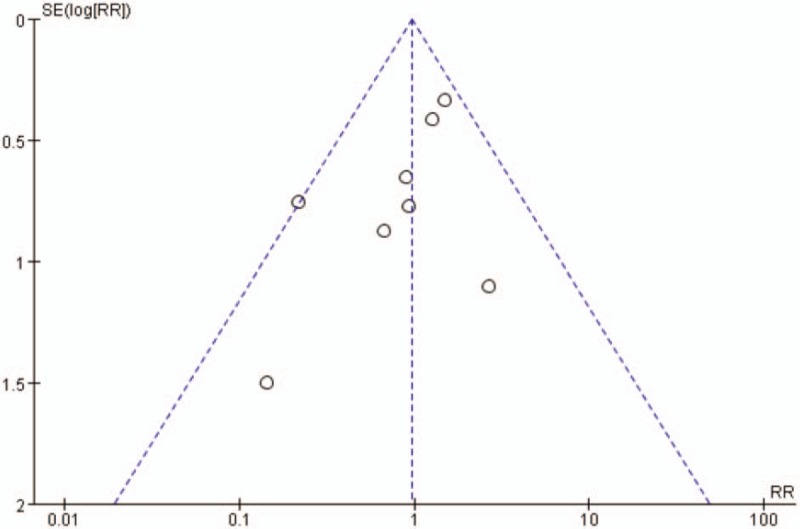
Funnel plot of publication bias test based on complications.

The funnel plot was drawn based on the index of complications, and the results showed that the distribution of each study in the funnel plot was asymmetrical, suggesting that there might be publication bias (Fig. 10). For the main outcome indicators with obvious heterogeneity, we removed individual studies one by one and then compared the statistical results among them, and the results showed good stability.

## 4 Discussion

The results showed that the barbed methods were superior to the traditional methods in terms of wound closure time, wound closure total cost and incidence of acupuncture injury. There was no significant difference in complications, suture breakages, knee society scores, and knee range of motion between the two methods.

In terms of wound suturing time; since the barbed suture can provide continuous suturing without repeated knotting, the suturing time can be shortened compared to conventional suturing. Our study showed that the use of barbed sutures shortened the suture time by 4.41 min compared to traditional sutures. Rubing et al^[29]^ performed a multicenter randomized controlled clinical trial showing that the use of barbed sutures can close the wound more quickly are comparable to traditional sutures. Rosenberg et al^[30]^ also supported this view. EIickmann et al^[31]^ have shown that differences in wound closure time may be due to differences in surgeon training levels, but they also acknowledge that a particular surgeon uses barbed sutures when compared traditional stitching is faster, these research reports are consistent with the results of my study. We found in the result of statistical, Smith et al^[19]^ and others have obvious heterogeneity study compared with other research, explore the reasons we found that compared to the rest of the study, Smith et al^[19]^ and pedestrian incision closure for the four layers suture (more than the rest of the study for three layer suture), and there are two layer suture in traditional suture group using continuous suture technique, interrupted suture more time consuming than continuous suture, so compared with other research extended the incision closure time, showing obvious heterogeneity. In addition, we carried out sensitivity analysis and compared the statistical results after eliminating individual studies one by one, and the results showed good stability. Studies have shown that postoperative infection may be associated with the duration of surgery, extending the exposure time increases the risk of postoperative infection, and increases the risk of infection by 9% every 15 min.^[32,33]^ Therefore, barbed stitching is a better choice for those who are looking for more effective stitching methods.

In terms of wound closure total cost; in this study, the total cost refers to material costs and the cost of operating room time, and our statistical results show that barbed stitching can save about $282.61 compared to traditional stitching. Zhang et al^[6]^ found that barbed stitching can reduce stitching time by 3.56 min compared to traditional stitching, saving $290.72. Maheswar et al^[34]^ also believe that the cost of traditional sutures is higher than that of barbed sutures ($82.59 vs $66.78). These reports are consistent with the results of this study. It is worth noting, however, that operating room costs vary from country to country, with the average operating expenses of 100 hospitals in the United States at about $62 per minute,^[35]^ but in China, the cost of operating room time is not charged. Therefore, Li et al^[36]^ suggest that the cost of barbed stitching is about $53.05 higher than traditional stitching, because we know that the cost of a single barbed suture is higher than that of traditional stitching materials. In addition, in terms of cost-effectiveness, we should also consider shortening the operation time in order to reduce patients’ exposure to narcotic drugs, which reduces the cost of narcotic drugs, more importantly, is safer for patients. At the same time, we should also consider operating room care costs and other costs, but all reports do not consider these aspects.

In terms of complications; according to previous studies, barbed sutures may cause more complications, some researcher believed that using the barbed suture method will increase superficial infection rate in total knee arthroplasty.^[37]^ Especially a report that the use of barbed suture to repair the failure of flexor tendon is an example.^[38,39]^ However, Vincent et al^[23]^ believe that barbed suture had significantly lesser wound complications, especially stitch abscess and dehiscence. Our results show that there is no significant difference in the incidence of postoperative complications between barbed suture and traditional suture and was consistent with Zhang's results.^[6]^ We believe that the causes of different complication rates in different studies are as follows:

1. Whether it is a barbed suture or a traditional suture, a variety of methods are used to suture the wound (Table 2), which results in the heterogeneity of the wound suture approach being inevitable, resulting in different results.
2. We believe that different clinicians may have different causes of complications due to different suture techniques.

In knee society scores and knee range of motion; good wound suture after total knee arthroplasty is essential for the recovery of joint function, because knee society scores and knee range of motion have different outcomes at different times after surgery, so we performed a subgroup analysis to compare differences between the two groups at different times. Our research results show that:

1. In terms of knee society scores, there was no significant difference between the barbed suture and the traditional sutured knee society scores after ≤6 weeks, although the barbed suture was superior to the traditional suture at ≤3 months, we believe that this difference is not clinically important.
2. In the knee range of motion, there was no significant difference in knee range of motion between the two groups after ≤6 weeks and ≥3 months, this result is consistent with the results of Alerander.^[25]^

However, given the low correlation between the two indicators, more studies are needed to demonstrate the reliability of the results, as well as long-term follow-up, to see if there are differences in long-term outcomes.In terms of suture breakages; previous studies have shown that due to the unique material structure of the barbed suture, the suture is prone to break at the barb. Malhotra et al^[24]^ suggest that when the wound is sutured, the barbed suture is more than the traditional suture, it is prone to breakage. But a cadaver studies have shown that barbed sutures can withstand higher tensions and periodic loads.^[38,39]^ Our results showed that although the barbed suture breaks more than the traditional suture, but the difference is not statistically significant. We believe that most of these suture breaks occur in the learning process of young physicians, and the accumulation of experience can reduce the incidence of suture fracture.

In the aspect of acupuncture injury; since the traditional suture needs repeated knotting, more instruments need to be transferred in the suture, thus causing an increase in the incidence of needle acupuncture injury in the suture.^[40]^ Our study shows that the application of barbed suture can significantly reduce the incidence of acupuncture injury, which is consistent with the results of Gilinland,^[26]^ because acupuncture injury may cause the spread of some infectious diseases, the application of barbed suture can be a good protection for medical staff.

Our research incorporates the high quality RCTs to date and demonstrates the value of the application of barbed sutures in primary total knee arthroplasty. But, we acknowledge that this study has some limitations:

1. This study only included English literature, and there is a risk of missing some research.
2. The study included in the study was short-term follow-up time after total knee arthroplasty and could not provide long-term efficacy comparison.
3. Due to the small number of articles included and insufficient sample size, we cannot make statistical analysis of other important indexes such as blood loss after operation.

Therefore, we need high quality, large sample studies to further demonstrate the safety and effectiveness of barbed sutures in the initial total knee arthroplasty application.

## 5 Conclusion

In the primary total knee arthroplasty, compared with the traditional suture, the application of barbed suture not only does not increase the incidence of wound complications, but also can shorten the operation time, save the cost of surgery, and can reduce the incidence of intraoperative acupuncture injury, which is worthy of clinical physicians promotion for the use.

## Author contributions

Conceived and designed this meta-analysis: Yaowen Qian, Kehu Yang, Pengbiao Li, Wenhui Zhang. Performed this study: Wenhui Zhang, Pengbiao Li and Peijing Yan. Analyzed the date: Wenhui Zhang, Pengbiao Li, Peijing Yan, Yanyan Wang, Jinlong Li, Shifang Guo, Jie Li, Zongru He. Contributed analysis tools/material: Wenhui Zhang, Pengbiao Li, Peijing Yan. Drafting the manuscript: Pengbiao Li, Wenhui Zhang.
